# Hospitals

**Published:** 1919-04

**Authors:** 


### Hospitals.

There was a time when pretty good homes were built without baths in them. If the writer is not mistaken the Governor's Mansion at Austin was built without a bath in it, and one was not added until many years later. Now the most humble cottages are equipped with bath rooms that are regarded as essential as doors and windows. Not many years ago hospitals were regarded as of use only to the very rich or the very poor. Only the rich it was thought could afford to pay for such a luxury as hospital treatment; the poor were sent to the hospitals because they had no other place to be treated. Now, the hospital is regarded as a public convenience both for physicians and for people of all classes who find it much better to be treated in a well equipped place than in the homes. It will not be many years before we will wonder how we ever got along without such institutions just as much as we now wonder how we were ever able to do without bath rooms in our homes.

